# Chronotropic Modulation of the Source-Sink Relationship of Sinoatrial-Atrial Impulse Conduction and Its Significance to Initiation of AF: A One-Dimensional Model Study

**DOI:** 10.1155/2015/496418

**Published:** 2015-07-01

**Authors:** Francesca Cacciani, Massimiliano Zaniboni

**Affiliations:** ^1^Department of Life Sciences, University of Parma, Parco Area delle Scienze 11A, 43124 Parma, Italy; ^2^Center of Excellence for Toxicological Research (CERT), Department of Clinical and Experimental Medicine, University of Parma, Via Gramsci 14, 43126 Parma, Italy

## Abstract

Initiation and maintenance of atrial fibrillation (AF) is often associated with pharmacologically or pathologically induced bradycardic states. Even drugs specifically developed in order to counteract cardiac arrhythmias often combine their action with bradycardia and, in turn, with development of AF, via still largely unknown mechanisms. This study aims to simulate action potential (AP) conduction between sinoatrial node (SAN) and atrial cells, either arranged in cell pairs or in a one-dimensional strand, where the relative amount of SAN membrane is made varying, in turn, with junctional resistance. The source-sink relationship between the two membrane types is studied in control conditions and under different simulated chronotropic interventions, in order to define a safety factor for pacemaker-to-atrial AP conduction (SASF) for each treatment. Whereas antiarrhythmic-like interventions which involve downregulation of calcium channels or of calcium handling decrease SASF, the simulation of Ivabradine administration does so to a lesser extent. Particularly interesting is the increase of SASF observed when downregulation *G*_Kr_, which simulates the administration of class III antiarrhythmic agents and is likely sustained by an increase in *I*_CaL_. Also, the increase in SASF is accompanied by a decreased conduction delay and a better entrainment of repolarization, which is significant to anti-AF strategies.

## Introduction

Atrial fibrillation (AF) is the most common cardiac arrhythmia, characterized by high morbidity and mortality; the mechanisms underlying its initiation, known to be complex and multifactorial, are still largely unexplained [1]. The most generally recognized causes of initiation and maintenance of AF are conduction abnormalities along interatrial accessory pathways [2, 3], abnormal interaction between sinoatrial node (SAN) cells and cardiac tissue nearby the insertion of pulmonary veins [4], increased fibrotic deposition within atrial tissue [5], electrical and calcium handling remodeling* per se* [6, 7] or secondary to atrial tachycardia (AT) [8, 9], and altered connexins ratio (Cx40/Cx43) leading to heterogeneity of conduction velocity [10, 11]. This complex scenario basically underlies two main pro-AF mechanisms, that is, triggered activity and reentry [12]. Furthermore, the crucial role of bradycardia in initiating and maintaining AF is well documented, either when associated with pathological conditions, like the sick sinus syndrome [13, 14], or brought about experimentally by cholinergic hyperactivation [15, 16]. Importantly, SAN spontaneous activity plays an active role in controlling atrial arrhythmias by its ability to terminate or convert atrial flutter to AF during cholinergic withdrawal [17].

A factor that makes the treatment of AF particularly complex is the paradoxical proarrhythmic effect of some of the most commonly used antiarrhythmic drugs. A typical case is the administration of Adenosine which, meant to terminate AT, frequently triggers AF by increasing potassium conductance via *I*_K,ACh_ [18]. Analogous adverse effect is commonly found with Dobutamine and with other antiarrhythmic agents, whose mechanism of action involves shortening of ECG Effective Refractory Period (ERP) and/or of atrial Action Potential Duration (APD) by increasing repolarizing potassium currents [18–21].

An increasingly adopted and promising bradycardic agent is Ivabradine (Iva), which slows heart rate without significantly affecting inotropy [22], and thus it is widely used in the treatment of angina [23, 24]. Unlike beta-blockers, Iva acts by directly closing *I*_*f*_ channels, the main responsible for cardiac membrane pacemaker depolarization [25]. Despite that, its administration is associated with a 14% increased risk of AF, when compared with other bradycardic agents [26], with the involved mechanism resembling that of the sick sinus syndrome [27].

Finally, we note that not only class IV antiarrhythmic drugs (*I*_CaL_ blockers) are occasionally associated with a higher risk of AF [28, 29], but also *I*_CaL_ downregulation is a common finding in conditions when AT precipitates to AF [30].

Most of the substrates leading to AF include decrease in heart rate suggesting a common, even if not exhaustive, underlying mechanism. Despite this fact, evidences explaining why bradycardia should favor initiation of AF have not been provided so far. The aim of the present simulation study is to explore, at the most elementary tissue level, that is, interaction within cell pairs and a one-dimensional strand, the role of pacemaker source, atrial sink relationship in establishing more or less safe impulse conduction under chronotropic conditions typically involved in the treatment of AF. Safety factor measurements are based on changes in intercellular coupling and in the SAN/atrial membrane surface ratio. Mathematical modeling allows quantification of such a factor for SAN-atrial drive under ion channels modulation, which can better direct pharmacological and clinical antiarrhythmic strategies.

## 2. Materials and Methods

### 2.1. Integrating Single Cell and Cell Pairs Action Potentials

All simulations reported in the present study have been performed by means of two mathematical models of rabbit cardiac action potentials (APs): the Severi et al. sinoatrial model [31] and the Lindblad et al. atrial model [32]. Both models were downloaded and recompiled in their MATLAB version by means of COR facility at http://cor.physiol.ox.ac.uk/. The “ode15s” solver built into the R2010b version of MATLAB (The MathWorks, Inc., USA) was used to integrate the models equations. All simulations were run on a PC with Intel Core 2, 3 GHz CPU. SAN-atrial electrical cell coupling was simulated by solving the following system of differential equations:

(1) $$\begin{array}{c} \frac{V_{m,\text{at}}-V_{m,\text{sa}}}{R_{J}}=S\left(C_{m,\text{sa}}\frac{{dV}_{m,\text{sa}}}{dt}+I_{\text{ion},\text{sa}}\right),\\ \frac{V_{m,\text{sa}}-V_{m,\text{at}}}{R_{J}}=\left(C_{m,\text{at}}\frac{{dV}_{m,\text{at}}}{dt}+I_{\text{ion},\text{at}}\right), \end{array}$$

where *R*_*J*_ is the global electrical gap junctional resistance (MΩ) between the SAN and atrial elements, *C*_*m*,sa_ and *C*_*m*,at_ are the electrical capacitance of the original sinoatrial and atrial models (32 and 100 pF, resp.), *I*_ion,sa_ and *I*_ion,at_ are the total ionic current (nA), and *V*_*m*,sa_ and *V*_*m*,at_ are the membrane potential (mV) in both models. The left term of both equations represents the electrotonic current (nA) flowing, through the *R*_*J*_ and with opposite sign, across SAN and atrial membrane, respectively. *S* is a scaling factor that allows the control of the size (membrane surface) of the SAN cell model. *S* = 2, for example, simulates electrical coupling of two parallel connected SAN cells firing synchronously, that is, a single SAN cell with twice the membrane surface and same channel densities, to the same atrial cell. *S* was made varying throughout all simulations between 1 and 20, in order to specifically investigate an (*S* and *R*_*j*_) region where the 3 pacing conditions (NP&ND, P&D, and P&ND) were all represented together with all the possible transitions between them. Sinoatrial AP waveforms, either in control or under any simulated intervention, were integrated starting with initial conditions taken after 120 s of spontaneous beating. Similarly, atrial AP waveforms were obtained by using initial conditions taken after 100 simulated electrically driven APs at a pacing cycle length (CL) intrinsic for the rabbit heart (355 ms).

### 2.2. Integrating AP Propagation in a One-Dimensional Cell Strand

Action potential propagation along a strand made of a variable number (*N*_SA_) of SAN cells and 10 atrial cells (Severi and Lindblad model, resp.), longitudinally connected with each other with an electrical resistance *R*_*J*_, was simulated by solving, for each *k*th cell, the following differential equation:

(2) $$\begin{array}{c} \frac{V_{k-1}-V_{k}}{R_{J}}-\frac{V_{k}-V_{k+1}}{R_{J}}=C_{m,k}\frac{{dV}_{k}}{dt}+I_{\text{ion},k}. \end{array}$$

Source-sink properties were investigated by varying *N*_SA_ between 1 and 10 and *R*_*J*_ from 5 up to 200 MΩ, step 5 MΩ. In some cases, in the last cell of the atrial side of the cable, a 2 ms suprathreshold current injection was simulated in order to elicit an AP propagated in atrial-SAN direction.

### 2.3. Simulated Experimental Conditions

#### 2.3.1. Autonomic Modulation of Rate

The effect of autonomic agonists acetylcholine (ACh) and isoproterenol (Iso) on the SAN AP model is built-in functions of the original Severi et al. formulation, and we refer to it [31] for the description of both treatments on ion channels, membrane transporters, and calcium dynamics.

#### 2.3.2. Modulation of the Membrane and Calcium Clocks

As in the Severi et al. original paper, we simulated the application of 3 *μ*M Iva, which corresponds to the block of 66% of *I*_*f*_ conductance [31, 33]. Since a univocal formulation for Ryanodine application in order to silence calcium clock [34, 35] is not provided for the Severi et al. model (whose CL barely changes after complete block of sarcoplasmic reticulum (SR) calcium release), we followed the indications by Maltsev and Lakatta [36] and simulated a Ryanodine-like (Rya^∗^) application by turning off SR calcium up-take and simultaneously downregulating SAN *G*_CaL_ by 34%, the aim being, as in all bradycardic treatments under study, to match the 28% CL prolongation found with Iva.

#### 2.3.3. Effects of Classes III and IV Antiarrhythmic Agents

The action of a class III antiarrhythmic agent like Dofetilide was simulated in single SAN cells and in cell pairs by a 74% *G*_Kr_ downregulation [37] and that of a typical class IV agent like Verapamil via 61.5% reduction of *I*_CaL_ conductance [38]. In cable simulations only 10% downregulation of both conductances was applied.

## 3. Results

Aim of the present study is to compare the effect of different simulated chronotropic interventions on the source-sink relationship between a SAN and an atrial rabbit AP model, either arranged in cell pairs or in a cells strand, in order to study differences in the electrical strength of the pacemaking source and discuss the corresponding significance to the initiation of AF.

### 3.1. Estimating the Strength of the Source

**Figure 1:**
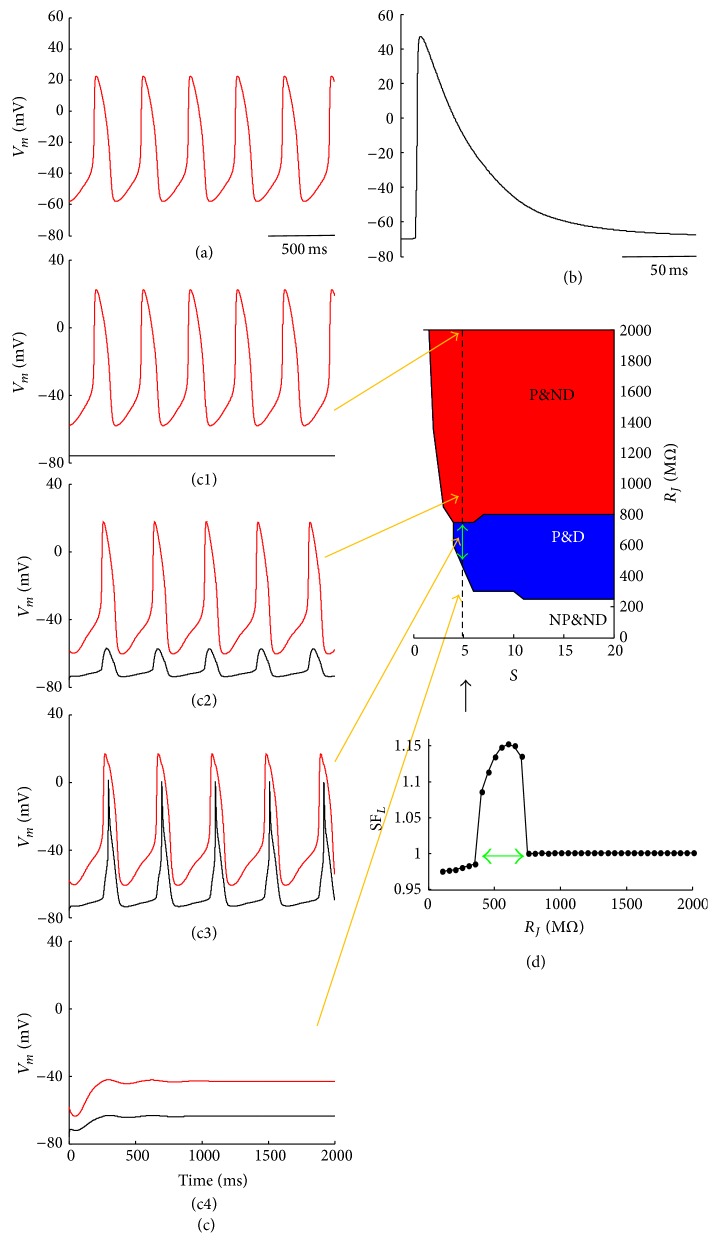
*SAN-atrial AP conduction.* (a) Severi et al. rabbit SAN simulated APs sequence. (b) Lindblad et al. rabbit atrial electrically driven AP, simulated at the physiological pacing rate of 355 ms. (c1–c4) Electrical coupling of the two AP models (SAN in red and atrial in black) according to equations system (1), for *S* = 5, and *R*_*J*_ = *∞*, 850, 700, and 300 MΩ, respectively. (d) Points of the (*R*_*J*_ and *S*) plan correspond to different conditions concerning SAN-atrial AP conduction: not pace and not drive (NP&ND, white), pace and drive (P&D, blue), and pace and not drive (P&ND, red). Lower inset: SF calculated with the Leon and Roberge formulation (see Section 3) for *S* = 5. The green double arrows label the same *R*_*J*_ windows in panel (d) and lower inset.

Figure 1(a) shows spontaneous firing of the original Severi et al. rabbit SAN AP model, whereas Figure 1(b) shows a single AP obtained by the Lindblad et al. atrial model by simulating a 2 ms depolarizing current injection at a physiological pacing rate. The two AP models were then electrically coupled as in System (1) in Methods. The dynamical behavior of the cell pair can be summarized in 3 different types of response: high values of *R*_*J*_ correspond to a condition when SAN pacing occurs but does not provide enough electrotonic current to drive the atrial cell at its own frequency (“pace and not drive,” P&ND, red in panel (d)). This is shown in panels (c1) and (c2): in (c1) *R*_*J*_ is set to be infinitely high, cells are practically uncoupled, and SAN cell displays its intrinsic spontaneous rhythm whereas atrial cell is quiescent at its resting potential (*V*_*r*_ = −75 mV). In (c2) *R*_*J*_ is high but small enough to allow depolarizing electrotonic current to flow across it and induce subthreshold depolarizations into the atrial model cell. By further decreasing *R*_*J*_, we find a window of its values where, despite the electrical load exerted by the more polarized atrial membrane, SAN cell keeps firing and provides enough electrotonic current to synchronously drive the atrial cell (“pace and drive,” P&D, blue in figure). Finally, when *R*_*J*_ is decreased below a certain value, the electrical load of the atrial cell prevails, preventing SAN firing and, with that, its own pacing (“not pace and not drive,” NP&ND, white in figure). It is found experimentally that these three conditions are not necessarily present for any cell pair but depend on the relative amount of membrane surface of the source and the sink cells [39]. This latter property is reproduced by the scaling parameter *S* of the first equation of System (1) reported in Methods. Thus the source-sink behavior of the SAN-atrial cell pair can be summarized in a graphic like that reported in panel (d), previously described by Joyner and van Capelle [40], and obtained here by varying *S* (step = 1) and *R*_*J*_ (step = 50 MΩ). Given an excitable pacemaking source, electrically coupled to an excitable resting sink, there is, in general, an *R*_*J*_ value separating NP&ND from P&ND or from P&D conditions. Also, only above a given value of *S*, P&D condition can take place (*S* > 4 in panel (d)). The ensemble of (*S* and *R*_*J*_) values which allow P&D condition fills the blue area, whose surface, as we will show, is a measure of the safety factor with which the SAN membrane can drive the atrial sink; we refer to the numerical value of such a factor as SASF. SASF surface scales dimensionally as MΩ. The conductance value of a single gap junctional channel (50 pS [41]) sets a nonlinear discretization for the *Y*-axes of Figure 1(d), which would allow us to convert the measured surface into a given number of gap junctional channels. Nevertheless, for the sake of the relative quantification of the strength of the source in different pacing conditions, our assumption of continuity for the *Y*-axes (and therefore for SASF) serves well our scope. The ability of an excitable current source to sustain a safe AP conduction is usually measured as safety factor (SF), and slightly different methods have been proposed in order to estimate it [42]. For each value of *S* we could derive the *R*_*J*_-dependency of SF, which we calculated according to the Leon and Roberge formulation:

(3) $$\begin{array}{c} {\text{SF}}_{L}=\frac{\int_{A}^{}I_{\text{ion}}dt}{\int_{B}^{}I_{m}dt}, \end{array}$$

with *I*_ion_ the ionic current and *I*_*m*_ the total membrane current flowing, respectively, across the atrial cell membrane and integrated over the time when they are negative (*A* and *B* time windows, resp.). In doing so, we found that, for each value of the scaling factor *S*, SF_*L*_ is greater than 1 only for *R*_*J*_ values falling within the P&D area (see representative example in lower inset of Figure 1(d)). SASF surface gives, in other words, a compact representation of source-sink properties when both *R*_*j*_ and membrane scaling (*S*) are subject to changes.

### 3.2. Chronotropic Interventions on the SAN Firing

**Figure 2:**
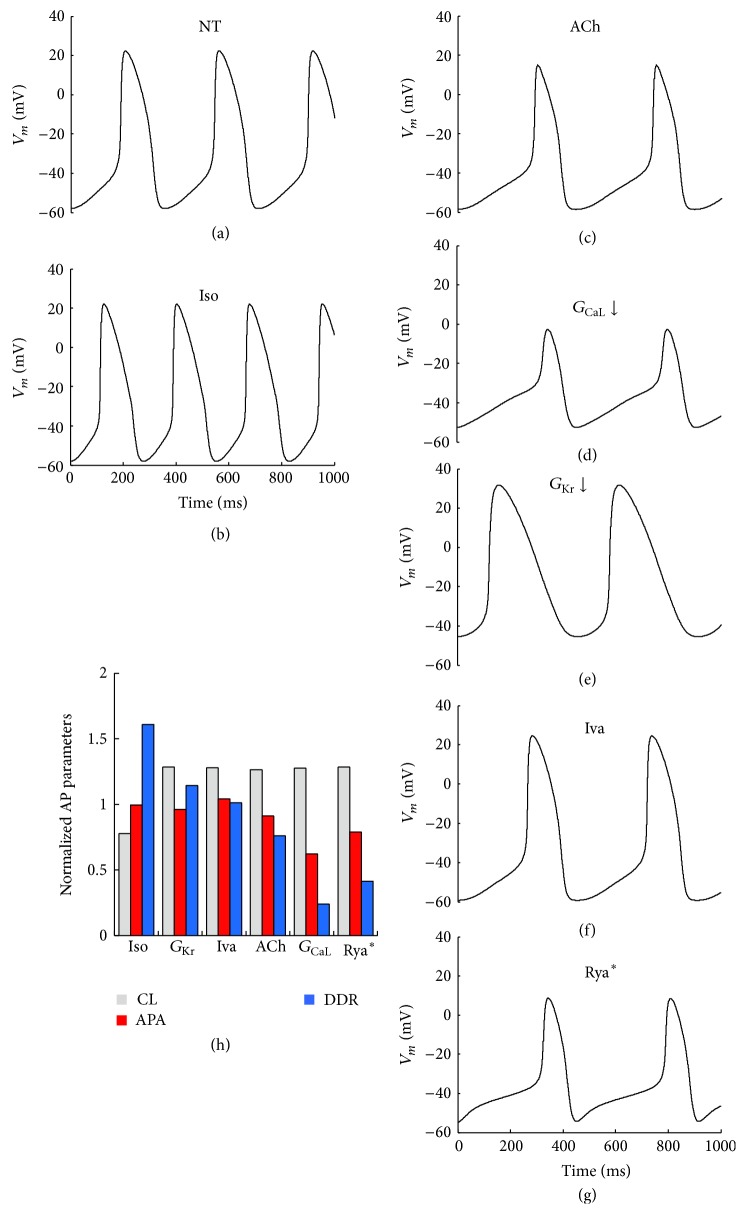
*Simulated chronotropic interventions on Severi *et al.* SAN AP firing*. (a–g) 1 s long APs sequences reported in their steady state conditions under the following treatments: physiological condition (NT), 1 *μ*M isoproterenol (Iso), 11 nM acetylcholine (ACh), 61.5% *G*_CaL_ reduction, 74% *G*_Kr_ reduction, 3 *μ*M Ivabradine (Iva), and 100% reduction of SR calcium uptake + 34% *G*_CaL_ reduction (Rya^∗^). (h) Treatments-induced changes in 3 AP parameters, as compared to their control values (NT).

In Figure 2 we summarize a number of different maneuvers (see Methods) we have performed on the SAN AP model in order to achieve increase (only one case) and decrease of its intrinsic beating rate. The simulated application of 1 *μ*M Iso leads to a 22% decrease in pacing CL by dramatically increasing the rate of diastolic depolarization (DDR, V/s) and leaving AP amplitude (APA, mV) unaltered (panels (b) and (h)). In contrast, the simulated application of 11 nM ACh leads to a 28% increase in CL (panel (c)), which is very close to that expected from the linear ACh-dose dependency for CL predicted by Rocchetti et al. in their study on real rabbit sinoatrial cells [43] and recently confirmed by our own study on guinea pig SAN cells [35]. We then simulated other interventions, like downregulating *G*_CaL_ by 61%, downregulating *G*_Kr_ by 74%, downregulating *I*_*f*_ conductance by 66% via simulation of 3 *μ*M Iva application [31, 33], and simultaneously downregulating SR rate of calcium uptake by 100% and *G*_CaL_ by 34% (panels (d)–(g)) in order to simulate Rya application (Rya^∗^, see Methods). In all these instances we fine-tuned parameters in order to achieve exactly the same bradycardic effect, that is, the same 28% CL increase obtained with ACh. Corresponding relative changes in APA and DDR are shown in the histogram of panel (h). Absolute values of AP parameters can be found in Table 1.

### 3.3. Relative Strength of the Sinoatrial Source

**Figure 3:**
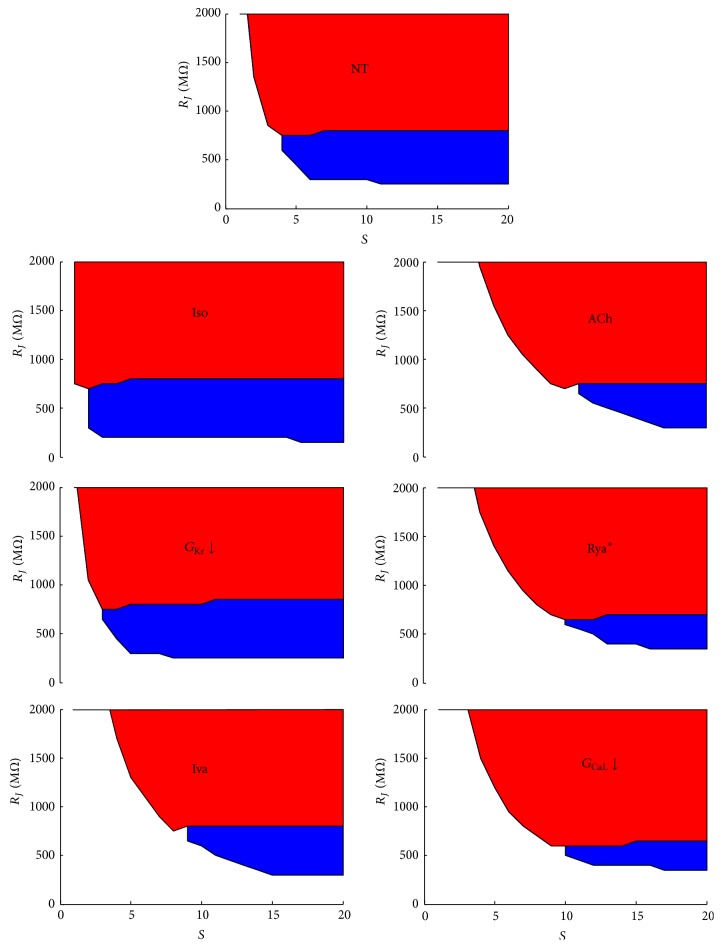
*Passive and active properties of SAN source-atrial sink. R*_*J*_ versus *S* plots, like that reported in Figure 1, were derived for each chronotropic intervention described in Figure 2. Iso corresponded to a 22% CL decrease, whereas all bradycardic maneuvers increased CL by 28%. Color code as in Figure 1. SASF values in MΩ were 8400 (NT), 11300 (Iso), 9550 (*G*_Kr_), 4850 (Iva), 3400 (ACh), 2950 (Rya^∗^), and 2550 (*G*_CaL_).

**Figure 4:**
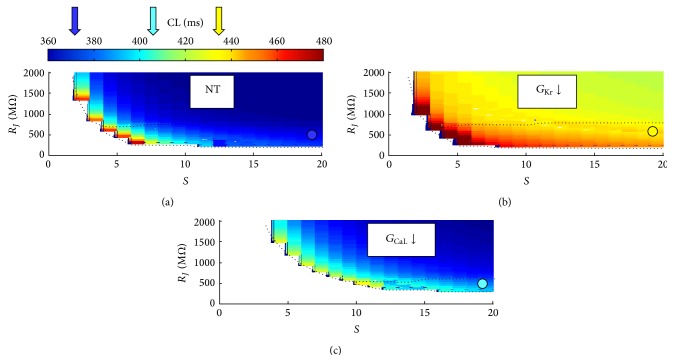
*CL changes related to source-sink properties*. Same representations of Figure 3 are reported for control (NT) conditions (a), 74% *G*_Kr_ downregulation (b), and 61.5% *G*_CaL_ downregulation (c), with the *Z*-axis representing a color contour map for corresponding CL values. Average CL values in P&D conditions are indicated with colored arrows pointing to the color bar on the right.

**Figure 5:**
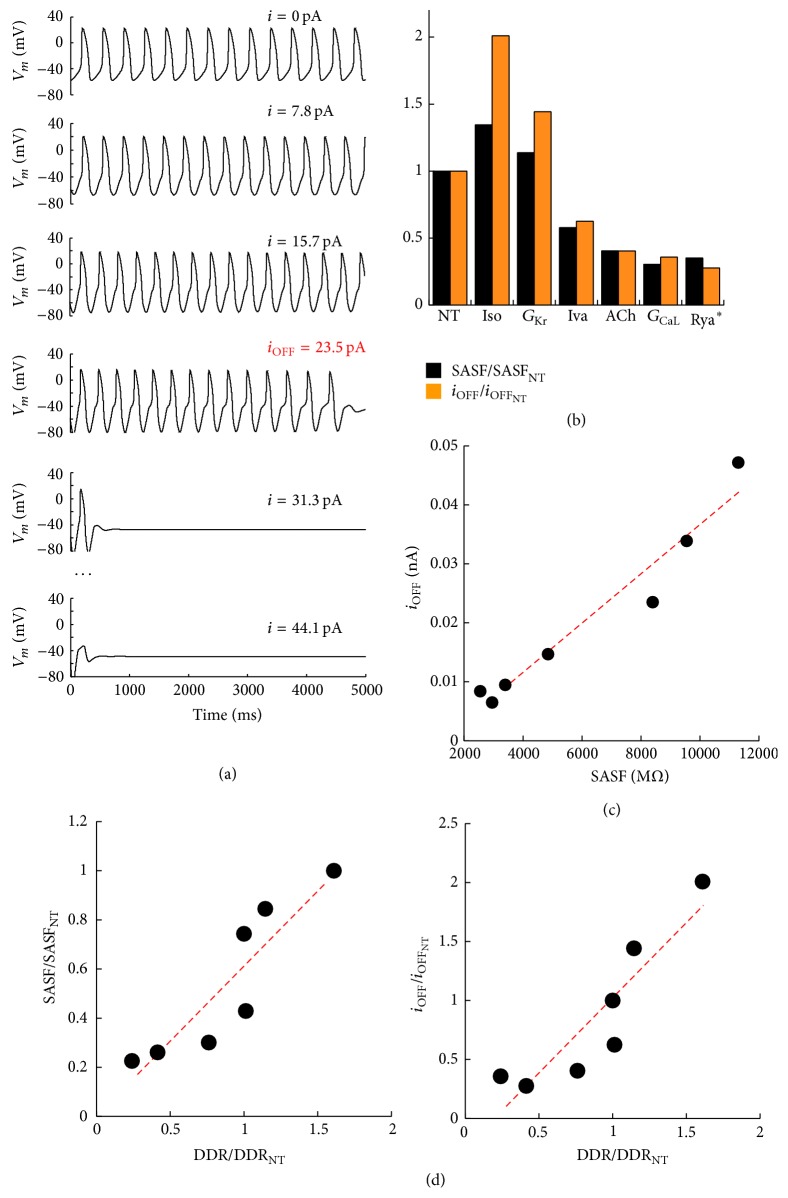
*Strength of the pacemaker as a current source.* (a) A simulation is shown where, during the NT steady state AP firing, a constant hyperpolarizing current of increasing amplitude was injected, until a critical value (in red) was reached, when firing stopped within 5 s. The same critical value was derived for all the treatments and reported in the histogram of panel (b), normalized to the value derived in NT (orange bars). In the same histogram, normalized values of SASF are reported as well (black bars). (c) Linear correlation between the absolute values of the two parameters reported in panel (b) (*R* = 0.97). (d) Normalized (to NT) values of SASF (left panel) and *i*_OFF_ (right panel) versus normalized values of DDR. In both instances a linear correlation (*R* = 0.90) was found.

For each one of the simulated SAN APs which underwent the chronotropic interventions described in Figure 2, we simulated electrical coupling with a Lindblad et al. atrial cell model by solving the equation system (1) reported in Methods and varying, in turn, coupling resistance *R*_*j*_ and scaling factor *S*, as explained for Figure 1(d). Each simulation resulted, as shown above, in NP&ND, P&D, and P&ND configurations, all summarized in the color panels of Figure 3. In the case of downregulation of *G*_Kr_ and *G*_CaL_, we also report in Figure 4, superimposed to the surface profiles shown in Figure 3 (black dotted lines), the color contour plots of the corresponding CL values. Our hypothesis that the P&D area of each graphic (SASF) is a measure of the strength of the SAN source is confirmed by the simulated experiment reported in Figure 5. In each beating SAN cell, taken in steady state conditions (after 120 s, see Methods), we simulated a hyperpolarizing constant current injection of increasing amplitude in order to find the amount of current needed to stop pacemaking within an arbitrarily chosen interval of time (5 s). Longer intervals were also tried and gave qualitatively identical results (not shown). The correlation between the constant current value needed to switch off pacemaking and the corresponding SASF value derived from Figure 3 clearly appears in the histogram of Figure 5(b) and is statistically quantified by the correlation analysis in panel (c) of the same figure. Whether we consider, for each treatment in the uncoupled condition and for the corresponding coupled configuration, the hyperpolarizing current needed to turn off pacemaking and the SASF value, respectively, we find that both parameters significantly correlate with DDR and with no other AP parameter (see panel (d)).

### 3.4. Transitory Changes in Electrical Coupling

**Figure 6:**
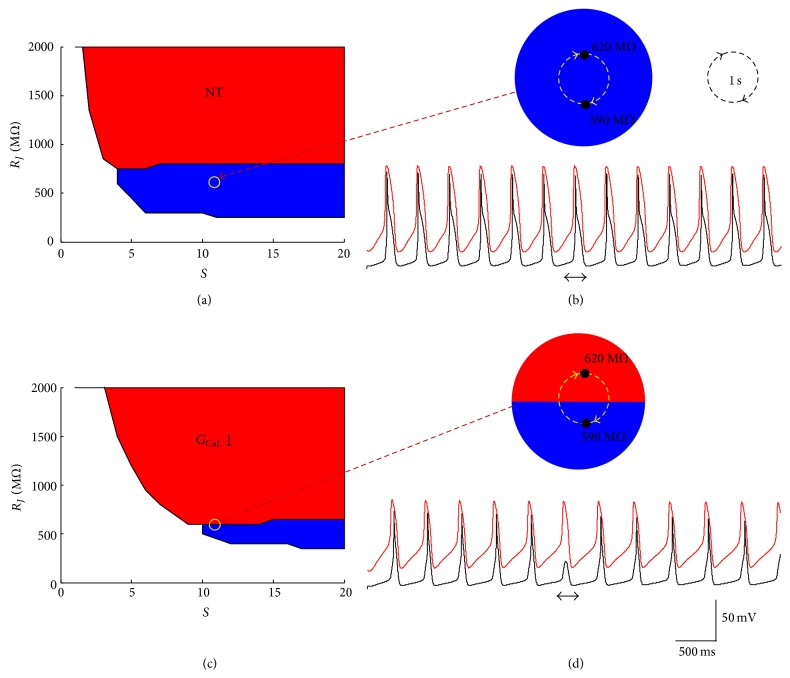
*Deviation from P&D set point*. SAN-atrial system is initially set to *S* = 11 and *R*_*J*_ = 590 MΩ (magnified detail in panel (b)) and transitorily displaced into *S* = 11 and *R*_*J*_ = 620 MΩ for 500 ms. (a, b) The transition is simulated under NT conditions, where it does not affect P&D dynamics (horizontal double arrow in lower panel (b)). (c, d) When the same transition is simulated under 61.5% *G*_CaL_ reduction, it transiently brings the system into the P&ND area (top panel (d)), leading to a single beat conduction block (horizontal double arrow in lower panel (d)).

We hypothesize here that the source-sink SAN-atrial system is set, in physiological conditions, at a given value of (*S*, *R*_*J*_), say (11, 590 MΩ); that is, 11 SAN cells are spatially arranged around a single atrial cell and are cumulatively coupled to it with a total of 34 gap junctional channels (assumed to have, as pointed out above, a single channel conductance of 50 pS). We further assume that none of the antiarrhythmic interventions that we simulate changes this set point (none of them has known effects on geometrical nor on gap junctional coupling). On the other hand, we know from the literature [10, 11] that AF is often accompanied by gap junctional remodeling. We show in Figure 6 what a transitory (500 ms) closure of only 2 gap junctional channels (yellow path in panels (a) and (c) and in the magnified details of panels (b) and (d)) is expected to cause on the safety factor of SAN pacing. Whereas in control conditions (NT) such slight junctional change leaves SAN-atrial intercellular conduction within the “safe” area (blue) of P&D behavior, the same modification transiently (horizontal double arrow in panel (d)) brings the *G*_CaL_ downregulated system into the P&ND region (red), where a single beat fails to be conducted from the SAN to the atrial membrane (bottom of panel (d)).

### 3.5. Relative Changes in Ion Currents for Each Treatment

**Figure 7:**
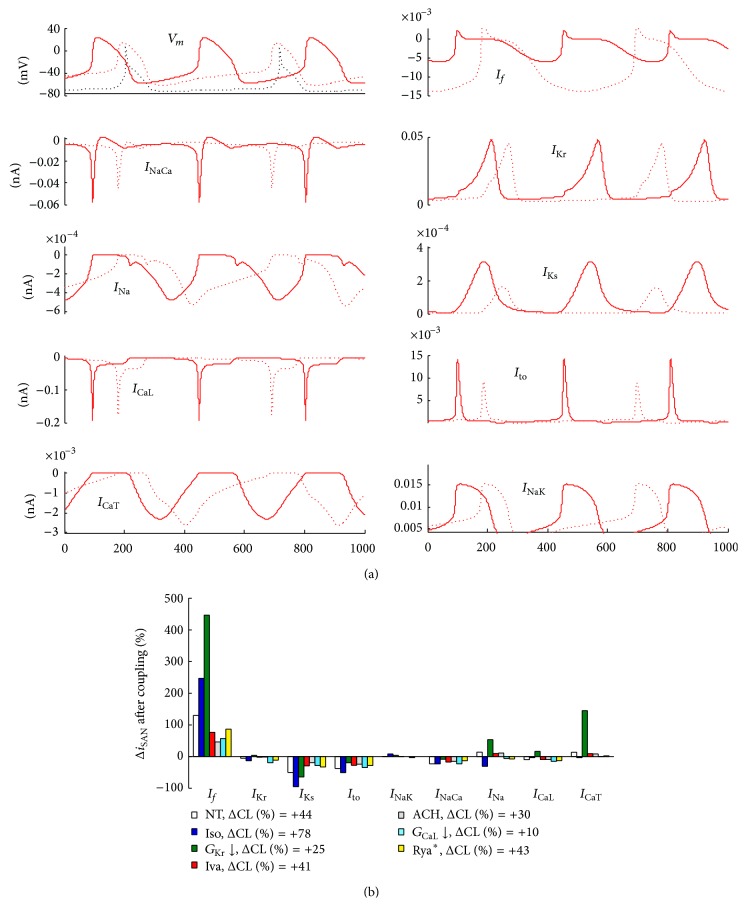
*Coupling-induced changes in SAN ion currents*. (a, top left panel) Time-course of SAN (red) and atrial (black) *V*_*m*_, before (solid line) and after (dotted line) electrical coupling with (*S*, *R*_*J*_) values chosen as reported in Results. The simulation corresponds to NT conditions. (a, remaining panels) SAN ion currents before (solid) and after (dotted) coupling. (b) Coupling-induced percent changes of the SAN peak ion currents under the 7 simulated treatments. Percent coupling-induced changes of the beating CL are reported in the inset.

**Figure 8:**
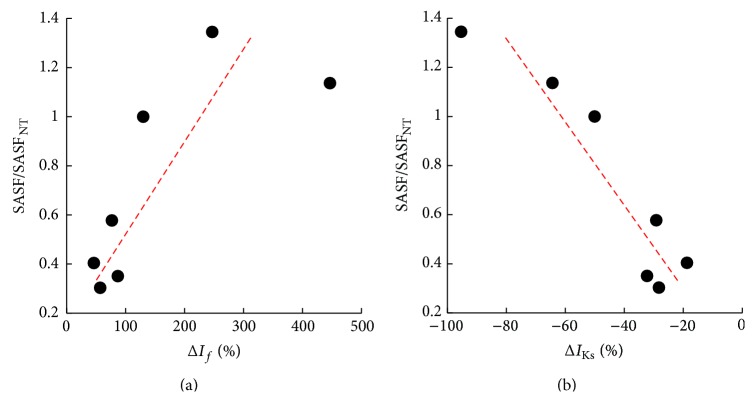
*Major ion currents contribution to SASF*. The value of SASF for each treatment, normalized to that in NT, correlates positively with coupling-induced percent changes in *I*_*f*_ (a) and negatively with coupling-induced changes in *I*_Ks_ (b) (values taken from histogram in Figure 7(b)).

In order to identify the ion currents primarily involved in determining the strength of the pacemaking source when coupled to an atrial sink, we performed a series of simulations like that reported in Figure 7(a) for the cell pair in NT. For each treatment, the minimum values of *S* and *R*_*j*_ (taken in this order), corresponding to the first permissive P&D configuration, were used to simulate cell coupling. Coupled (dotted lines) and uncoupled (solid lines) AP waveforms are reported for both cells (SAN in red and atrial in black) in the top left panel. The other panels represent the main SAN ion currents in the uncoupled and coupled conditions. The histogram in panel (b) represents corresponding percent coupling-induced changes in peak ion current of the SAN AP for the 7 treatments under study.  Figure 8 shows how SAN-atrial conditions characterized by a larger “safety factor” correspond to a larger increase in *I*_*f*_ when passing from uncoupled to coupled conditions (panel (a)). Similarly, they correspond to a larger decrease of *I*_Ks_ when coupled (panel (b)). Correlation coefficients were 0.77 and 0.93, respectively. Positive significant correlations were also found for *I*_CaL_ and *I*_NaK_, though involving much smaller changes (data not shown).

### 3.6. Conduction Delay and Entrainment of Repolarization

We have shown, up to this point, that the area of the blue surfaces in Figure 3 measures the safety factor SASF associated with spread of pacemaker activity from SAN to atrial cells. We have also observed that SASF is different among bradycardic interventions which lead to the same decrease in SAN beating rate. We also wanted to test how safety factor of pacemaking is going to affect AP conduction delay and entrainment of repolarization in our cell pair model. We did that for identical coupling conditions, that is, for SAN and atrial cells coupled with *S* = 14 and *R*_*J*_ = 550 MΩ, which correspond to a P&D state for all treatments.

**Figure 9:**
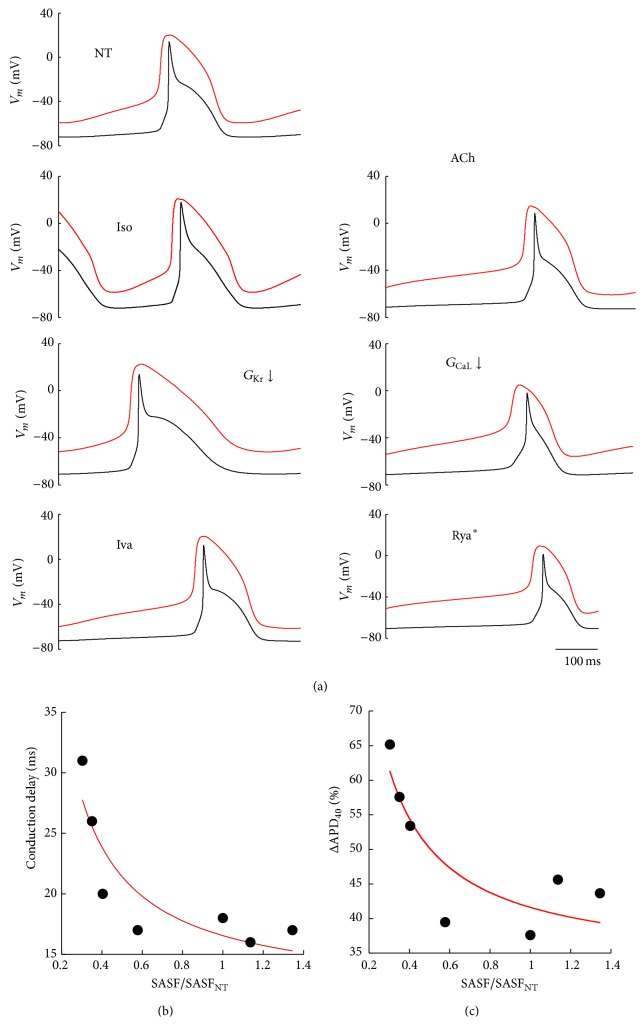
*Conduction delay and entrainment of repolarization*. (a) A single conducted beat (SAN in red and atrial in black), measured in steady state conditions and for *S* = 14 and *R*_*J*_ = 550 MΩ (see Section 3), is shown for all simulated treatments. (b) Delay between the time-to-peak of the first *V*_*m*_ time derivative for the source and sink APs is reported against normalized (to NT) values of P&D area. (c) Same representation for the delay between APD_−40 mV_ measured in the 2 APs for each treatment.

Coupled conditions were simulated for 5 s and the last conducted APs reported in Figure 9(a). Panel (b) shows how AP conduction delay decreases as safety factor increases, being their relationship well fitted (*R* = 0.89) by a hyperbolic function. Same for relative APD_−40 mV_ difference between SAN and atrial APs (*R* = 0.87) (panel (c)), where APD_−40 mV_ measures the time lapse between the peak of the initial *V*_*m*_ time derivative and the time when *V*_*m*_ reaches the value of −40 mV. Very similar results (data not shown) were found when *S* and *R*_*J*_ were chosen in order to roughly match the center of the P&D area for each treatment. In other words, a more robust pacemaker source not only guarantees a much higher safety factor for the spread of excitation but also leads to a shorter AP conduction delay and a better entrainment of repolarization as well.

### 3.7. Simulations of a Linear SAN-Atrial Cell Strand

**Figure 10:**
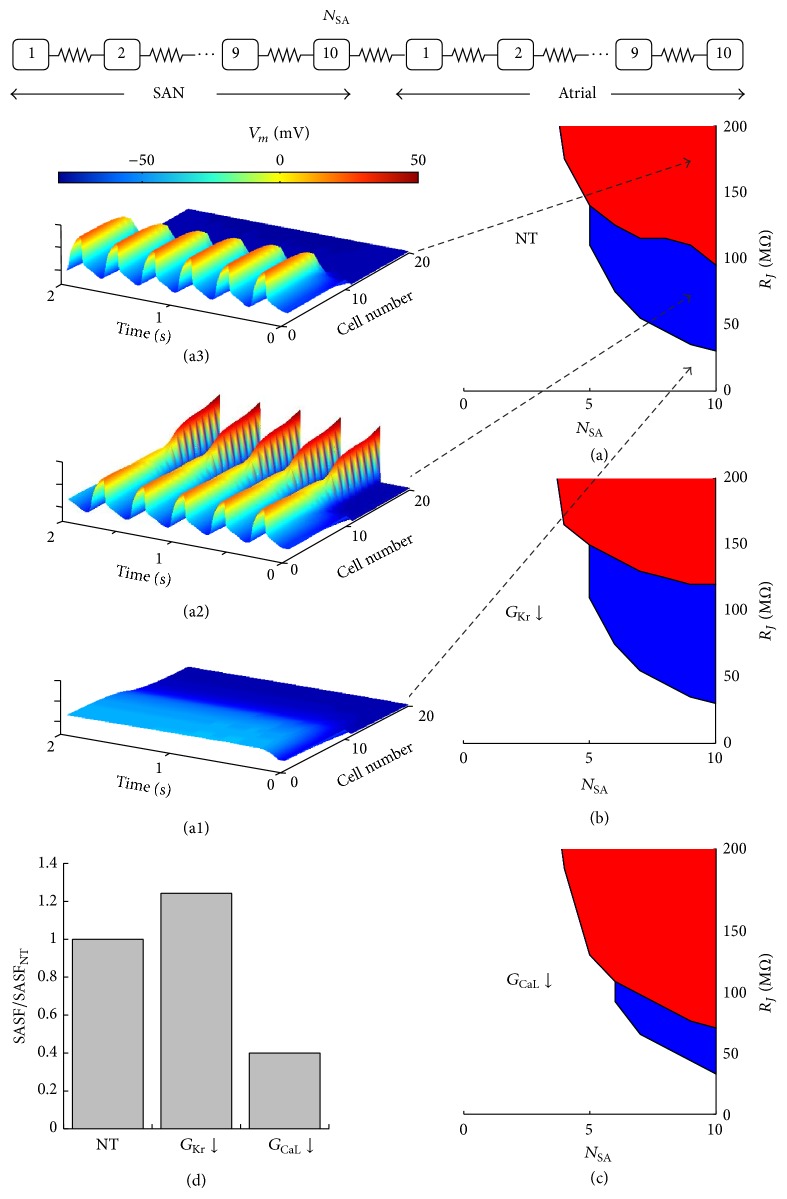
*AP propagation in a SAN-atrial cell strand*. A chain made of a variable number (*N*_SA_, ranging between 1 and 10) of SAN cells and 10 atrial cells intercellularly connected with an *R*_*J*_ ranging from 5 up to 200 MΩ was simulated and reported as a scheme in the top panel. (a–c) Source-sink properties measured as in cell pairs simulations (see Methods) are reported for control conditions (NT), 10% *G*_Kr_ downregulation, and 10% *G*_CaL_ downregulation. (a1–a3) Three-dimensional representations of NP&ND, P&D, and P&ND conditions for NT. (d) Normalized (to NT) values of SASF (blue areas in a–c) as measured for the 3 conditions.

In order to test whether SASF evaluation as a measure of source strength could be applied also in a cable-like AP propagation, we performed simulations on a SAN-atrial chain (see scheme, top panel of Figure 10) of cells, electrically connected with a variable junctional resistance (see Methods). For small *R*_*J*_ values, the more polarized atrial side of the cable completely depressed spontaneous firing in the left SAN side (NP&ND condition in Figure 10(a1) and white area in panel (a)). As *R*_*J*_ increased, and in analogy with what is observed for cell pairs reported in Figure 3, spontaneous APs were generated in the SAN side of the cable and conducted to the atrial side (P&D condition in Figure 10(a2) and blue area in panel (a)). A further *R*_*J*_ increase prevented APs generated in the SAN side from being conducted to the atrial side (P&ND condition in Figure 10(a3) and red area in panel (a)). The same is shown in panels (b) and (c) of Figure 10 in the case of 10% downregulation of *G*_Kr_ and *G*_CaL_, respectively. These results confirm, in a cable-like model, the effects reported above on cell pairs, where *G*_Kr_ downregulation increases and *G*_CaL_ downregulation decreases the strength of the source, that is, the safety factor for AP conduction (see also Figure 10(d)).

**Figure 11:**
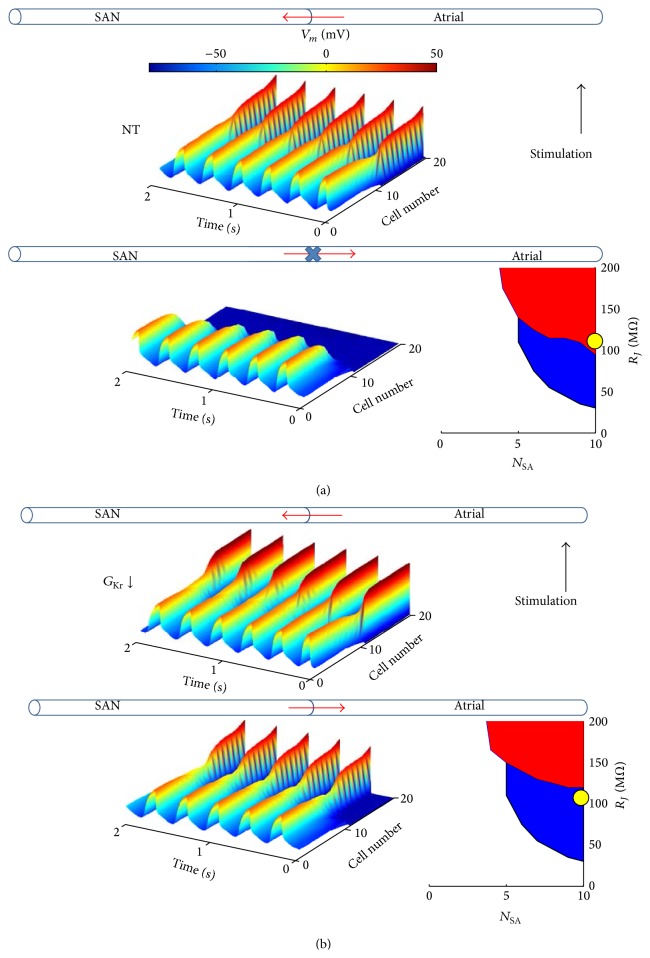
*Unidirectional block*. (a) In control (NT) conditions, and for cable properties defined by *N*_SA_ = 10 and *R*_*J*_ = 110 MΩ (yellow dot in the inset. Note that it falls into the red P&ND area), AP conduction succeeds in atrial-SAN direction, when atrial side electrical stimulation is simulated, but fails in SAN-atrial direction (no stimulus applied); see also colored three-dimensional representations. (b) When *G*_Kr_ downregulation is applied in the same cable conditions (yellow dot in the inset. Note that now it falls into the blue P&D area), AP conduction succeeds in both ways.

Given the relevance of unidirectional block (UB) in the initiation of AF, we tested for it the cable-like model described in Figure 10, with the additional aim of relating UB to source-sink properties as measured with SASF surfaces. UB developed when AP propagation was simulated in control conditions for *N*_SA_ = 10 and *R*_*J*_ = 110 MΩ (yellow circle in the SASF representation); that is, conduction failed when the SAN side of the cable was let free of beating at its own intrinsic frequency (CL = 341 ms) but succeeded when the end of the atrial side of the cable was electrically paced at the same frequency (Figure 11(a)). This was indeed expected, since the yellow circle is located into the P&ND (red) region of the graph. As we have shown in Figure 10 and reported here in the inset of panel (b), *G*_Kr_ downregulation extends the P&D (blue) area, which now includes the yellow circle. Accordingly, conduction becomes permissive also in the SAN-atrial direction, making *G*_Kr_ downregulation effective, in this case, also in removing UB.

## 4. Discussion

The present modeling study aims to investigate, at the cellular level, the source-sink SAN-atrial relationship in conditions which are critical for the development of AF. We first define a graphical representation which quantifies the safety factor for impulse conduction from SAN to atrial cell membrane into a single parameter (SASF). We then use SASF in order to compare identical bradycardic effects on SAN firing, which follow pacemaker autonomic regulation or antiarrhythmic-like treatments targeting AF.

Representations of the source-sink properties of SAN-atrial electrical coupling in graphics like those of Figures 1 and 3 have been previously described by Joyner and van Capelle [40], who showed that some degree of electrical uncoupling is an essential design for proper SAN-atrial conduction. Several other simulation studies have been performed on this same issue [44–46], though not addressing its potential relevance for the initiation of AF. Basically it has been demonstrated that some degree of SAN to atrial interdigitation improves reliability of conduction and that the critical junctional resistance allowing AP entrainment in SAN-atrial cell pairs can easily fall within the GΩs range. Though the geometry of SAN is far to be fully elucidated and the possible arrangement of mutual intercellular coupling still needs to be clarified, nevertheless it is assumed that SAN ability to electrically drive the larger atrial volume is based on the presence of different cell types within its interior [47, 48], on a complex gradient of gap junctional distribution [49, 50], and on some favorable intercellular geometry [47]. This latter is achieved by interdigitation of different cell types [46, 50], which will likely result into a many-to-one SAN-atrial cells connection. Graphics (Figures 1(d) and 3) reporting *R*_*J*_ versus *S* (number of SAN cells coupled to a single atrial cell) compactly summarize these properties, by defining, for each *S*, the *R*_*J*_-range that allows SAN membrane to electrically drive the atrial cell (P&D blue area). The same type of representations can be applied also to cable-like AP propagation (Figures 10 and 11).

Autonomic modulation, which is known to shift the leading pacemaker site within the SAN [51], is involved into the initiation and maintenance of AF [52]. Particularly, cholinergic stimulation is indicated as one of the main factors for the initiation of spontaneous AF, even though the exact knowledge of underlying mechanism is unknown [52]. Our simulations of cholinergic (equivalent to 11 nM ACh) and adrenergic (equivalent to 1 *μ*M Iso) modulation of the Severi et al. SAN AP model show that the two treatments lead to a large decrease and increase of SASF, respectively (Figures 3 and 5(b)), which is likely underlying the increased propensity to AF under cholinergic hyperstimulation [53] and related to the dramatic differences in coupling induced-Δ*I*_*f*_ in the two instances (Figure 7(b)). Indeed, the correlation between SASF and Δ*I*_*f*_ (Figure 8(a)) suggests that coupling-induced changes in *I*_*f*_ play a major role in determining SASF.

Recent studies have attributed the molecular mechanism of SAN rhythm to the interplay between two clocks, one involving the hyperpolarization activated cation current *I*_*f*_ (membrane clock) [54] and the second attributable to activation of the electrogenic NaCa exchanger by spontaneous SR releases of calcium (calcium clock) [36]. The two clocks have been shown to possess different intrinsic dynamic properties, which can contribute to the physiopathology of the heart rhythm [35]. Thus, it is of interest to investigate the source-sink changes induced into the SAN when the two clocks are separately downregulated in order to obtain the same bradycardic effect. Whereas both maneuvers lead to a decrease in SASF, we note that the simulated application of Iva reduces SASF to a lesser extent when compared to Rya^∗^ (Figures 3 and 5(b)). If, from one hand, the downregulation of the membrane clock appears to be safer than that of the calcium clock, on the other hand the Iva-induced reduction in SASF might be the mechanism underlying the slight increase in AF development found when Iva is used, as it is frequently, instead of beta-blockers in the treatment of ischaemic diseases [26].

Sinus node dysfunction is often associated with initiation and maintenance of AF [13], and an electrotonically weaker SAN, even in the absence of structural, electrical, or junctional remodeling, is more prone to transient SAN blocks, leading to a substrate favoring arrhythmias. This is shown, for instance, in the case of *I*_CaL_ downregulation (Figure 6), where even a very rapid and transient 5% increase in *R*_*J*_, ineffective in physiological conditions (panel (b)), brings the system out of the P&D area, leading to a 1-beat failure in SAN-atrial conduction (panel (d)). A comprehensive study of the SASF associated with pharmacological treatments of AF is beyond the scope of the present study. We limit our analysis to two ion channels modulatory effects involved in the action of classes III and IV antiarrhythmic drugs, like Dofetilide and Verapamil, respectively, known to decrease *I*_Kr_ and *I*_CaL_ conductance [38, 55]. Furthermore, since our aim was to compare bradycardic effects of the same extent (28% reduction of CL), we did not try to match, in our simulations, the therapeutic doses clinically administered for each one of these agents.

*I*_CaL_ downregulation, which results from class IV antiarrhythmics administration, is also a common finding in conditions when atrial tachycardia develops into AF [30]. It is interesting, at this regard to observe that a decrease in *G*_CaL_ brings about a dramatic reduction of SASF both in cell pairs (Figures 3 and 5(b)) and in cable propagation (Figures 10(c) and 10(d)), which might explain the transition to AF due to (1) a decreased safety factor for AP conduction, (2) an increased conduction delay, and (3) a diminished control in the entrainment of repolarization (Figures 9(b) and 9(c)). This is in agreement with the experimental findings of Hondeghem and Hoffmann in isolated rabbit hearts [29], and with documented Verapamil-induced SAN asystole [28] as well. An opposite effect on SASF is brought about in SAN-atrial cell pairs by the downregulation (−74%) of *G*_Kr_ in order to simulate the action of pure anti-*I*_Kr_ class III antiarrhythmic agents, like Dofetilide and Nifekalant [56, 57]. In this case, the decrease of *G*_Kr_ leads to (1) an increase in SASF, likely mediated by the coupling-induced greater increase in *I*_*f*_ and in *I*_CaL_ (Figures 7(b) and 8(a)), (2) a decrease in conduction delay, and (3) a better entrainment of repolarization, all synergic with a reduced proneness to develop AF. Furthermore, the larger coupling-induced reduction in *I*_Ks_ (Figures 7(b) and 8(b)) is going to reduce the likelihood of AF initiation by further increasing APD [58].

The role of *I*_CaL_ in granting safety of conduction can be summarized as follows. The depolarizing (negative) *I*_ion_ exceeding the depolarizing (negative) electrotonic *I*_*m*_ (see (3)), which makes AP spread safe (SF_*L*_ > 1) or, in other words, makes the source sink system fall into the P&D area, is almost entirely due to the contribution of *I*_CaL_, which is far the larger depolarizing current in SAN membrane. When this current is reduced, SASF gets reduced as well (Figure 3) and, with it, do the strength of the SAN source and the beating frequency (bottom panel of Figure 4). When, on the opposite, *I*_CaL_ increases, like during *G*_Kr_ reduction (see Figure 7(b)), the safety of conduction increases as SASF does (Figure 3); beating frequency decreases (middle panel of Figure 4) more than it does under *G*_CaL_ downregulation.

Finally, in the cable simulation of Figure 11, SASF calculation predicts UB for a given set of passive parameters (*N*_SA_ and *R*_*J*_, yellow dots in the insets) in control conditions, and its removal after *G*_Kr_ downregulation following SASF increases. Unidirectional block, together with decrease in conduction velocity, is a well-recognized cause of reentry, which, in turn, as mentioned above, is one of the two main mechanisms underlying initiation of AF [12]. The fact that, as we show, decreasing *G*_Kr_ prevents UB induced by electrical uncoupling, might point to an unexplored property of class III antiarrhythmic agents, which are usually mainly described for their AP prolonging action.

To summarize, it is accepted that the successful SAN drive of the atria is based on a combination of active and passive electrical properties, and on a given geometrical arrangement between the two cell types within the SAN node and at its border [47]. The three factors determine a set point which, in physiological conditions, belongs to the P&D area of the graphic representations of Figure 3. The surface of such area can be used to quantify (SASF) the strength of the SAN as an electrotonic source. We find that (1) para- and orthosympathetic modulations exert opposite effects on SASF, (2) class III antiarrhythmic agents increase it and class IV decreases it, and (3) the same bradycardic effect obtained by separately downregulating the membrane and calcium clock corresponds to a smaller and larger SASF reduction, respectively.

## 5. Conclusions

Among the several conditions leading to AF, the role of safety in SAN-atrial AP conduction in terms of source-sink relationship has not been previously explored. Our simulation study shows that such relationship should be taken into account, together with usually recognized active, passive, and geometrical factors, in order to reconstruct the mechanism underlying the initiation and maintenance of AF. Particularly, a parameter like SASF compactly describes the strength of SAN source and thus might draw light on further SAN modeling and on the mechanism of action of antiarrhythmic agents.

## Limitations of the Study

Despite the critical role that the morphological structure of SAN-atrial arrangement, including the complex interaction between several recognized cell types [47, 59, 60], is going to play into the atrial transition to fibrillation, such information could not possibly be considered in our simulations, given the still incomplete and sparse literature on the subject, and the much simpler geometry of our simulation setting. On the other hand, our simplified approach is meant to provide a more general background on SAN source-atrial sink properties, which can be improved as the underlying structure will be elucidated. A limitation of the Severi et al. SAN AP model is the lack of sensitivity of beating rate to reduction of SR calcium release. Thus, as mentioned above, in order to simulate Rya application, we followed a procedure already in use by other authors [36]. Finally, unfortunately the measure of SASF cannot possibly be pursued in any thinkable experimental preparation, which confines its use to the theoretical* in silico *explanation of experimentally observed phenomena.

## Figures and Tables

**Table 1:** Sinoatrial AP parameters under chronotropic interventions. Beating parameters were measured after 100 s of simulated spontaneous firing, with AP waveform in its steady state conditions. Cycle length (CL), maximum diastolic potential (MDP), upstroke *V*_*m*_ value (*V*_peak_), action potential amplitude (APA), action potential duration as measured at −40 mV (APD_−40 mV_), diastolic depolarization rate (DDR), maximum value of the first time derivative of AP (*dV*/*dt*_max_), and threshold value as take-off potential (TOP) are reported.

	CL	MDP	*V*_peak_	APA	APD_−40 mV_	DDR	*dV*/*dt*_max_	TOP
	(ms)	(mV)	(mV)	(mV)	(ms)	(V/s)	(V/s)	(mV)
NT	355	−58.01	22.32	80.33	125.7	0.79869	7.22	−18.42
Iso	276	−57.97	22.04	80.01	127.5	1.2862	11.13	−25.02
*I*_Kr_	456	−45.51	31.71	77.22	274.7	0.91447	5.75	−19.08
Iva	454	−59.19	24.53	83.72	132	0.80923	6.05	−18.46
ACh	449	−58.58	14.81	73.39	103.6	0.60802	7.79	−19.32
*I*_CaL_	453	−52.54	−2.567	49.973	84.4	0.19244	1.47	−20.74
Pup	456	−54.89	8.612	63.502	96.2725	0.33101	3.76	−20.8
